# Pleomorphic Adenoma Presenting as an Atypical Nasal Mass in a 26-Year-Old Female

**DOI:** 10.1155/2020/3726397

**Published:** 2020-08-27

**Authors:** Racheal Hapunda, Chibamba Mumba, Owen Ngalamika

**Affiliations:** ^1^ENT Unit, Surgery Department, Adult University Teaching Hospital, University of Zambia School of Medicine, Zambia; ^2^Pathology and Microbiology Department, Adult University Teaching Hospital, University of Zambia School of Medicine, Zambia; ^3^Dermatology and Venereology Division, Adult University Teaching Hospital, University of Zambia School of Medicine, Zambia

## Abstract

Pleomorphic adenoma (PA) is a salivary gland tumor that may rarely occur in the nasal cavity. It can be a clinical diagnostic dilemma in many instances due to many possible differential diagnoses. We report the case of a 26-year-old female who presented with a 3-year history of a right nasal growth associated with ipsilateral nasal blockage, nasal pain, and rhinorrhea. Radiological image showed a mild enhancing lesion in the right nasal cavity. The patient underwent a lateral rhinotomy with wide excision of the mass. Histopathological exam was consistent with PA. Nasal PA is a rare entity and should be suspected as a diagnosis for intranasal tumors.

## 1. Introduction

Pleomorphic adenoma (PA) is the most prevalent salivary gland tumor in both children and adults, and it has a 2 : 1 female-to-male ratio. The parotid gland is the most common site accounting for 70-80% of the cases followed by the submandibular gland at 10% [1, 2]. About 5-10% of the cases occur in minor salivary glands found in the lips, palate, nasal cavity, paranasal sinuses, larynx, trachea, and the thyroid gland [3–6].

PA rarely arises in the respiratory tract, but when it does, the nasal cavity is the commonest site [7]. About 80% of nasal pleomorphic adenomas are detected in the nasal septum, while the remaining 20% are found in the lateral wall in spite of mucous and serous glands being confined to the lateral nasal wall [7].

Here, we report the case of a 26-year-old female who presented with nasal PA.

## 2. Case Presentation

**Figure 1:**
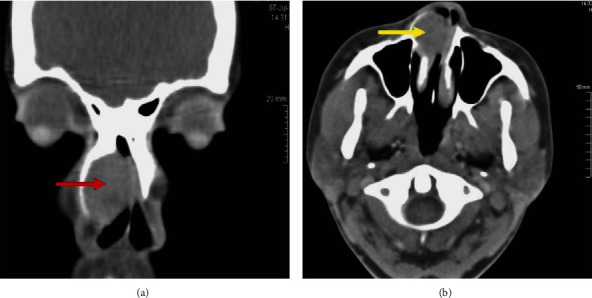
Computer tomographic scan images of the paranasal sinuses. (a) Coronal cut showing a homogenous image filling the right nasal cavity (red arrow). (b) Axial cut with contrast showing enhancing lesion compressing the right lateral nasal bone and nasal septum without bone destruction (yellow arrow).

A 26-year-old female presented with a 3-year history of a right nasal growth associated with ipsilateral nasal blockage, nasal pain, and rhinorrhea. On examination, she was noted to have a right-sided nasal bridge deformity. On anterior rhinoscopy, the right nasal cavity had a hard mass emerging from the inferior turbinate abutting on the nasal septum. The mass was completely filling the nasal cavity and obstructing the middle meatus. Nasal decongestion with a topical decongestant was unsuccessful. Baseline routine laboratory investigations and HIV tests were normal (Table 1). HIV testing is mandatory in our setting due to high HIV prevalence. Computer tomography scan showed a round homogenous mild enhancing lesion (measuring about 20 × 28 mm) in the right nasal cavity compressing the right nasal bone and nasal septum without bone destruction. The rest of the sinuses were normal (Figure 1). An initial impression of a benign nasal mass was made. The patient was then booked for a lateral rhinotomy and excision of nasal mass.

### 2.1. Intraoperative Findings

We found a round, well-encapsulated mass measuring 40 mm × 30 mm arising from the inferior lateral wall encased by the inferior turbinate mucosa. The inferior turbinate appeared to be thinned by the mass. The mass was excised with its pedicle and part of the inferior turbinate.

**Figure 2:**
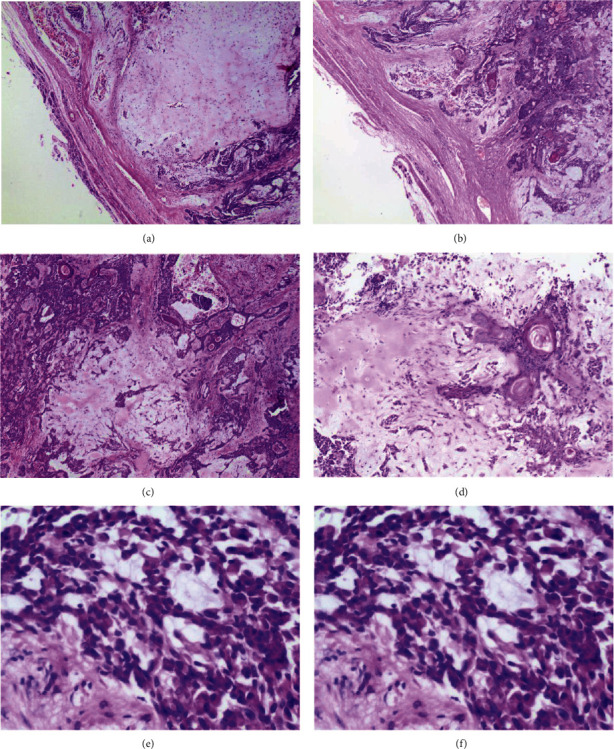
Pleomorphic adenoma, showing cytoarchitectural features characteristic of this tumor. Capsule and a focus of chondroid stroma (×100) (a) and a variegated growth pattern (×100) (b) are seen. Tubules containing eosinophilic material (×200) (c) and foci of squamous differentiation (×200) (d) are seen. A myxoid background in which plasmacytoid hyaline cells (modified myoepithelial cells) are seen (×400) (e and f).

Gross examination revealed a greyish brown tissue measuring 40 mm × 30 mm × 30 mm with foci of bony elements and a capsule on cross-sectioning. The mass was then sent for histopathology (Figure 2), which revealed respiratory-type epithelium underneath which was a tumor with partial encapsulation and a variegated appearance comprising a chondroid (hyaline cartilage) and myxoid matrix in which were tubules lined by an inner layer of ductal cells and an outer layer of myoepithelial cells. Plasmacytoid hyaline cells with eccentrically placed nuclei and abundant eosinophilic cytoplasm (modified myoepithelial cells) were seen in sheets and islands and as single cells in the myxoid stroma. Foci of squamous differentiation were readily evident. Malignant elements were not seen. A diagnosis of pleomorphic adenoma was then made.

The patient was discharged on postoperative day 3, and she recovered without any complications. On follow-up 2 years later, the patient had adequate nasal patency with no recurrence of the nasal mass.

## 3. Discussion

Nasal pleomorphic adenoma commonly presents as a nasal mass with unilateral nasal blockage which slowly progresses and may cause external nasal deformity associated with pain [8], which was the case in our patient. The patient may also have occasional epistaxis [9], although our patient had no epistaxis.

The clinical differential diagnoses include several lesions, most of which were polypoids, such as angiofibroma, osteoma, squamous cell carcinoma, adenocarcinoma, lymphoma, and melanoma. Recommended imaging options include CT scan and MRI. CT scan awards the clinician the chance to assess bone involvement or erosion.

Although it was not done in our patient, preoperative fine needle aspiration of the mass is advisable as a cytological diagnosis guides surgical approach, given the wide spectrum of clinical differential diagnoses. Histologic examination of PA classically demonstrates myoepithelial and epithelial cells, some with duct formation, and mesenchymal or stromal elements [10].

PAs in the nasal cavity may be misdiagnosed as an aggressive epithelial tumor due to high cellularity and a significantly reduced stromal component, features less common in tumors found in major salivary gland sites [2]. In our case, this diagnostic pitfall was not encountered as the tumor demonstrated clear areas of chondromyxoid stroma.

Increased cellularity gives the nasal PA a biologically aggressive nature. Malignant transformation is seen in 2-6% of all salivary gland tumors [11]. Between septal and lateral wall PA, the former has a higher chance of malignancy transformation into carcinoma, e.g., pleomorphic adenoma with metastasis into bone, lungs, liver, and lymph nodes [12]. Our patient had a benign PA without evidence of malignant transformation.

Nasal PA has a lower rate of recurrence compared to PA of the parotid gland at 10 and 50%, respectively [13]. An average recurrence period of 11.9 years for PA has been seen in some cases [14]. The ideal treatment for nasal pleomorphic adenoma included wide local excision along with periosteum and involved bone. Surgical approaches depend on site and size, and they include intranasal excision, lateral rhinotomy, midfacial degloving, and transpalatal approaches. In our case, we used lateral rhinotomy with a wide local excision of the tumor including partial resection of the inferior turbinate.

## 4. Conclusion

Nasal pleomorphic adenoma is a rare entity and should be suspected as a diagnosis for intranasal tumors. Wide local excision with clear margins and close follow-up postoperatively is necessary due to the potential risk of local recurrence.

## Figures and Tables

**Table 1:** Baseline laboratory investigations.

Parameter	Result
White cell count	6.6 × 10^9^/L
Hemoglobin	13.1 g/dL
Platelet count	247 × 10^9^/L
Erythrocyte sedimentation rate	8 mm/hr
HIV antibody test	Negative
